# The Poet

**Published:** 1891-05

**Authors:** Richard E. Burton


### THE POET.

He's not alone an artist weak and white
O'er-bending scented paper, toying there
With languid fancies fashioned deft and fair,
Mere sops to time between the day and night.
He is a poor torn soul who sees aright
How far he fails of living out of the rare
Night visions God vouchsafes along the air
Until the pain burns hot, beyond his might.
The heart-beat of the universal will
He hears, and, spite of blindness and disproof,
Can sense amidst the jar a singing fine.
Grief-smitten that his lyre should lack the skill
To speak it plain, he plays in paths aloof,
And knows the trend is starward, life divine.

RICHARD E. BURTON, in *The Century.*

